# An update on diagnosis and therapeutics for type-2 diabetes mellitus

**DOI:** 10.6026/97320630019295

**Published:** 2023-03-31

**Authors:** Babu Shyamaladevi, Ipsita Dash, Ramya Badrachalam, Madhan Krishnan, Arjunkumar Panneerselvam, Sadhana Undru

**Affiliations:** 1 Faculty of Allied Health Sciences, Chettinad Hospital and Research Institute, Chettinad Academy of Research and Education, Kelambakkam-603103, Tamil Nadu, India; 2 Department of biochemistry, Saheed Laxman Nayak Medical College and Hospital, Pujariput, Koraput, Odisha- 764020; 3 Department of Biochemistry, Sri Manakula Vinayagar Medical College & Hospital, Madagadipet, Kalitheerthalkuppam, Puducherry - 605107, Puducherry, India; 4 Department of Pharmaceutics, Arulmigu Kalasalingam College of Pharmacy, Krishnankoil, Tamil Nadu, India; 5 Department of Mental Health Nursing, Kims college of Nursing, KIMS & RF, Amalapuram, East Godavari district, Andhra Pradesh, India

**Keywords:** Type 2 diabetes mellitus, Diagnosis, Oral drugs, Insulin Secretagogues, Insulin sensitizer and dipeptidyl peptidase-4 inhibitors

## Abstract

Type-2 Diabetes mellitus is a common metabolic disorder. It is combined with co-morbidities, such as obesity, hyperlipidemia, hypertension and cardiovascular disease which taken together, comprise the 'Metabolic Syndrome'. This disease causes crucial morbidity and mortality at considerable expense to patients, their families and society. Different categories of drugs such as insulin secretagogues, insulin sensitizers, alpha-glucosidase inhibitors, GLP-1 agonists, DPP4 inhibitors, dual PPAR agonists and others are used for its management. Therefore, it is of interest to highlight the recent advances in diagnosis and therapeutics used in the treatment of type-2 diabetes mellitus. The classical and online-literature were used to compile data for this study. This includes the electronic search engine such as Scopus, Google Scholar, Sci Finder, PubMed and Web of Science. Data shows that there are different families of oral and injectable drugs at hand for the treatment of T2DM. Hence, we need to develop a novel, safety and effective agents that will improve the quality of life of T2DM patients, considering effectiveness and durability of lowering blood Glucose, risk of hypoglycemia and diabetes complications.

## Background:

Diabetes mellitus (T2DM) is one among the very common metabolic disorder and is associated with co-morbidities such as obesity, hyperlipidemia (increased VLDL triglycerides and decreased HDL cholesterol), hypertension and cardiovascular disease which taken together, comprise the 'Metabolic Syndrome'. It is characterized by relative insulin insufficiency results in hyperglycemia [1]. Behavioral studies states that agitating levels of satiety - related hormones in circulation in children leads to obesity and diabetes [2]. It is a long standing degenerative disease, results in relatively Specific long-term complications affecting the eyes (retinopathy), kidneys (nephropathy), and peripheral and autonomic nervous systems (neuropathy) accounting for vision loss, end-stage kidney disease and amputations than any other disease. Among various types, Type-2 diabetes affects more people and common symptoms seen in type-2 diabetes are increased thirst, frequent urination, tiredness, slow-healing wounds, recurrent infections and tingling or numbness in hands and feet [3]. This disease causes crucial morbidity and mortality at considerable expense to patients, their families and society. International Diabetes Federation (IDF) 2019 report is stating that around 463 million people worldwide are suffering from this disease and the prevalence is predicted to cross the figure of 700 million by the year 2045. China and India are the two main epicenters of the developing T2DM global epidemic in Asia. Although the current global epidemic is mostly driven by bad diet and modern lifestyle, genetic predisposition and early developmental variables (including intrauterine exposures) also play a part in an individual's susceptibility to T2DM later in life [4,5].

## Types of diabetes Mellitus:

Type-1 diabetes (also known as insulin-dependent, juvenile, or childhood-onset diabetes) is characterised by a partial or total lack of insulin owing to autoimmune death of insulin-secreting beta cells in the pancreas and the requirement for daily insulin injection. The most important risk factors for type- 1diabetes are family history, age, and genetics. The core cause of type 1 diabetes is unknown, and it is not preventive or treatable given current understanding. Excessive urine and thirst, persistent hunger, weight loss, eyesight problems, and weariness are all symptoms [6].Type-2 diabetes (also called as non-insulin dependent or adult onset diabetes) accounts for 90 % or more of all diabetes cases worldwide and is caused by the body's poor utilization of insulin. A number of main risk factors for type 2 diabetes exist. Diabetes risk factors include a family history of the disease, ethnicity, physical inactivity, smoking, and obesity. Gestational diabetes (GDM) arises during pregnancy and is a transient disease that increases the risk of type 2 diabetes in the long run. It is induced by insulin-blocking substances generated by the placenta. Family history, bad eating, race, being fat, pre-diabetes, and past, unexplained stillbirths are all risk factors [6].

## Global picture of Type-2 diabetes mellitus:

Asia is the epicenter of the global type-2 diabetes epidemic, with India and China leading the way. Diabetes mellitus affects around 463 million people aged 20 to 79 worldwide, with the number anticipated to climb to 578. 4 -3 million by 2030 and 700 million by 2045, ac cording to the International Diabetes Federation. The number of people with type-2 diabetes is increasing in most countries, and 79 percent of individuals with diabetes live in low- and middle-income countries. One out of every five people over the age of 65 is affected by it. A total of 374 million people are at risk of developing type-2 diabetes. It caused 4.2 million deaths and is estimated to cost USD 760 billion per year (10 percent of total spending) by 2030, rising to USD 825 billion by 2030 and USD 845 billion by 2045. China (116.4 million), India (77 million), and the United States of America (31 million) are the countries with the highest number of diabetic people (aged 20-79 years) and are expected to remain so in 2030, with Pakistan (19.4 million at present) expected to increase to 36 million by 2045, surpassing the United States of America and moving to third place by 2045 [4].

## Complications:

The diabetic patient with years of uncontrolled hyperglycemia has numerous vascular problems, which are classed as micro and macro vascular complications, affecting small and major vascular/blood arteries or both. Diabetic complications may be both debilitating and life-threatening. The mechanism by which vascular disorder develops includes: (a) the formation of glycation end products by glycosylation of tissue proteins; (b) superoxide production; (c) endothelial dysfunction caused by activating protein kinase C- signaling, which increased vascular permeability; (d) sorbitol accumulation within tissues; (e) dyslipidemias and hypertension; (f) arterial micro thrombosis; and (g) impaired vascular [3].

## Advances in Diagnosis:

Blood glucose measured in fasting state or after an oral glucose tolerance test has been used to diagnose type-2 diabetes. More recently, HbA1c levels have been recommended for the diagnosis of diabetes and pre-diabetes [7]. It reflects mean levels of glucose integrated over the life span of the protein. The HbA1c assays are now standardized and are a reliable index of average glucose levels over the preceding 8 to 12 weeks [8, 9]. Genetics and Metabolomics; Type-2 diabetes is a polygenic disorder. Nearly, 100 genes or genetic regions are concerned in type 2 diabetes. Whether new information about genetic risk factors can increase the identification of persons at high risk is unclear. In comparison to the use of easily measurable demographic and clinical factors like age, body mass index, systolic blood pressure, and fasting glucose and lipid levels, studies that have looked at the role of genetic profiles in the identification of risky individuals haven't demonstrated a significant additional benefit. In future, genotyping is going to play a useful role differentiating subtypes of polygenic disorder with different pathophysiological mechanisms and may facilitate to individualize the treatment of type 2 diabetes by distinctive persons a lot of likely to retort to specific treatments [10, 11]. The metabolomic studies revealed that the high circulating levels of hexoses, branched-chain amino acids, aromatic amino acids, phospholipids and triglycerides were associated with the incidence of pre-diabetes mellitus and T2DM. The metabolomics derived indices enable statistically significant improvement in the prediction of T2DM risk beyond the use of traditional risk factors. The prognostic value and the specificity of those metabolic fingerprints and their clinical utility haven't been established, but they may complement genetic markers [12, 13].

## New diboronic acid for the electro hydrodynamic monitoring of glucose:

Novel design of dicationicdiboronic acid structure (DBA2+) having excellent precise affinity (Kd ≈ 1 mM) towards glucose. It changes the pKa of DBA2+ from 9.4 to 6.3. This change facilitates the detection of glucose at physiological pH. Conductimetric method is used to detect the releasing levels of proton from DBA2+which is directly proportional to glucose concentration in physiologically ranges from 0 - 30 mM. This result suggested that, simple molecular structure of DBA2+ molecule used for non-enzymatic and conductimetric determination of glucose concentration. The author found that DBA2+molecule have precise selectivity and affinity to glucose with sufficient water solubility, and also have potency to change pKa upon glucose binding at physiological pH. More over other sugars does not have significant interference in this study. Finally this method was used to overcome the lack of selectivity in synthetic small molecule glucose -sensing methods and lack of stability in enzymatic glucose - sensing methods [14].

## Type-2 diabetes management:

**Figure 1:**
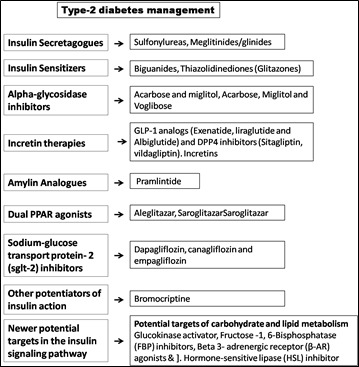
Type 2 diabetes management

Modification of lifestyle, including weight loss, increasing bodily activity and adopting a healthy diet, remains one of the first-line strategies for the management of T2DM. In addition, lifestyle programs oral hypoglycemic drugs are used.15The drugs used treating T2 DM can be divided into different categories such as insulin secretagogues, insulin sensitizers, alpha-glucosidase inhibitors, GLP-1 agonists, DPP4 inhibitors, Dual PPAR agonists etc (Figure 1).

## Insulin secretagogues:

Drugs that primarily stimulate the secretion of insulin, known as insulin secretagogues, include sulfonylureas: The proposed mechanism of action of the sulfonylureas includes (i) augmentation of insulin release from pancreatic β cells and (ii) potentiating of insulin action on its target cells. Tolazamide, chlorpropamide, gliclazide, tolbutamide are the earlier generation sulfonylureas. Glibenclamide (Glyburide) Glipizide, gliclazide, glimepiride are the newer generation sulfonylureas. The disadvantage of sulfonylureas is hypoglycemia and weight gain.16b) Meglitinides/glinides: This is another class of secretagogues that are similar to sulfonylureas in their mechanism of action but lack the sulfonic acid-urea moiety products. Nateglinide, repaglinide are fewer chances of hypoglycemia and useful in diabetic patients who have an inconsistent daily schedule with long gaps between meals. The undesirable effects are dyspepsia, weight gain and arthralgia [17,18].

## Insulin sensitizers:

Drugs that sensitize tissues (primarily liver and adipose tissue) to the action of insulin named as insulin sensitizers.

## a) Biguanides:

Phenformin has been discontinued due to its development of lactic acidosis in patients with coexisting liver or kidney disease. Metformin is used usually as first medicine, based on its efficacy in lowering glycemia, long history of use, demonstrated safety and tolerability, and other characteristics including the absence of hypoglycemia, associated weight loss, and low cost [19].

## b) Thiazolidinediones (Glitazones) (TZDs):

These agents sensitize peripheral tissues to insulin by binding to a nuclear receptor called peroxisome proliferators-activated receptor-gamma (PPARγ) [20].

Troglitazone was introduced first which caused acute liver failure. So its usage was withdrawn. The next 2 TZDs, Pioglitazone and are used as monotherapy, or combination with sulfonylureas, metformin, and insulin. Many scientifical Studies revealed that rosiglitazone was associated with increased risk of CVD [21] and pioglitazone with increased bladder cancer risk [22,23].Common side effects of TZDs' are weight gain, peripheral edema and macular edema, congestive heart failure and bone loss which limit their use.

## Alpha-glycosidase inhibitors:

Drugs that principally affect absorption of glucose include alpha-glucosidase inhibitors: Acarbose and miglitol are competitive inhibitors of intestinal brush border alpha-glucosidases and potent inhibitors of glucoamylase, a-amylase and sucrase, thus delaying the absorption of carbohydrates and reduce postprandial glycemic excursion. Acarbose, Miglitol and Voglibose are widely used in the management of type 2 DM. Miglitol is structurally similar to glucose, is absorbable and is similar to acarbose in terms of its clinical effects. Miglitol should not be used in renal failure since its clearance is difficult. The common adverse effect is flatulence, loose stools and liver enzyme rises [16,19].

## Incretin therapies:

Incretin therapies are the recent strategies which include GLP-1 analogs (Exenatide, liraglutide and Albiglutide) and DPP4 inhibitors (Sitagliptin, vildagliptin). Incretins are GIT hormones, produced in response to incoming nutrients that contribute to glucose homeostasis. GLP-1 agonists: It augments insulin release in response to ingested glucose and suppresses inappropriately high glucagon values which in turn put down hepatic glucose output [24]. It also shrinks the rate of gastric emptying, thus promoting satiety, resulting in the turn down caloric intake and weight slash [25]. It has been reported that it may preserve β cell reserves. It has adverse side effects such as nausea, vomiting, diarrhea, weight-loss, necrotizing and hemorrhagic pancreatitis [26].DPP4 inhibitors (Dipeptidyl Peptidase 4 Inhibitors): It acts slowing the breakdown of GLP-1 analogs. It works by enhancing the sensitivity of β cells to glucose, which causes enhanced glucose- dependent insulin secretion. Treatment with GLP-1 analogs and DPP4 inhibitors showed a sustained reduction in HbA1c and weight gain. The adverse effects are a headache, increased sweating, cough, nasopharyngitis, and constipation [24]. Other DPP4 inhibitors in Phase-III clinical trials are Linagliptin, anagliptin and dutogliptin [27].

## Amylin Analogues:

Amylin secretion is diminished in patients with diabetes which involved in the suppression of endogenous glucagon production, reduces postprandial hepatic glucose production and induces satiety. Pramlintide is a synthetic analog of amylin used to treat T2DM has adverse effects such as hypoglycemia and nausea [28].

## Dual PPAR agonists:

Saroglitazar is used for diabetic dyslipidemia. Phase II trial for Aleglitazar is completed [29, 30]. PPARs, nuclear receptors, involve in fatty acid metabolism. PPAR α agonists such as fibrates decreases the levels of triglyceride and increased the levels of HDL where as PPAR γ agonists such as thiazolidinediones reduces blood glucose levels. Dual PPAR (α and γ) agonists plays a role in both reduction of triglyerides and blood glucose and increasing HDL. The only dural α/γ PPAR/agonist available in is Saroglitazar. These medications have cardiac and renal toxicity.

## Sodium-glucose transport protein- 2 (sglt-2) inhibitors:

SGLT2 is expressed almost exclusively in the proximal tubule of the kidney. Inhibition of SGLT2, and thus inhibition of renal glucose reabsorption, has the potential to reduce hyperglycemia in patients with diabetes mellitus. Empagliflozin, Canagliflozin and Dapagliflozin, is commonly used SGLT2 inhibitors. It also has some limitations due to its adverse effects which include hypotension, amputations, bladder cancer and expand the incidence of urinary tract infection (UTI). Tofogliflozin (20-40 mg) is anewdrug, still in phase –III trial which has been developed by Chugai Pharmaceutica and approved in Japan for Type-2 diabetes mellitus, as either monotherapy or combination with other oral Antihyperglycemic agents [31].Remogliflozinetabonate the gliflozin class of drug which has been studied at doses up to 1000 mg for the treatment of nonalcoholic steatohepatitis (NASH) and type-2 diabetes mellitus. It was discovered by the Japanese company Kissei Pharmaceutical and commercially first lunched in India in may 2019 by Glenmark [32,34]. Its phase -IIb clinical trials for T2DM published in 2015 in which theyfound reductions in glycated hemoglobin and generally well tolerated [35].

## Other potentiators of insulin action:

Bromocriptine is a dopamine D2 receptor agonist. It has long been known to improve insulin sensitivity and glycemic control in T2DM. Bromocriptine as monotherapy or an adjunct to other antidiabetic agents has reduced HbA1, triglyceride and some cardiovascular events [36].

## Newer potential targets in the insulin signaling pathway:

This includes a) Insulin receptor activators: Pharmaceutical interventions aimed at mimicking insulin's effect and augmenting IR function may prove beneficial. Vanadate analogs are used that in higher doses frequently causes unwanted side effects including abdominal discomfort, diarrhea and nausea. b) Protein tyrosine phosphatase inhibitors (PTP-1b): Protein tyrosine phosphatase 1B (PTP1B) is thought to function as a negative regulator of insulin and leptin signal transduction. c) Glycogen synthase kinase-3(GSK-3b) inhibitors: Glycogen synthase is the rate-limiting step in glycogen synthesis and that is inactivated by GSK-3b.GSK- 3b inhibitor favors the synthesis of glycogen which is beneficial for the management of T2DM that is under pre-clinical stages [37].

## Potential targets of carbohydrate metabolism:

## a) Glucokinase activator:

The possible concern is increased hepatic glycogen, lipid deposition in liver and muscle [38], Piragliatin - phase 2 trail.

## b) Fructose:

1, 6-Bisphosphatase (FBP) inhibitors: Decrease hepatic glucose production, (Phase-II- MB-O7803).c) Glycogen phosphorylase (GP) inhibitors: Ingliforib is discontinued in the phase-II trail [37].

## Potential targets of lipid metabolism:

Beta 3- adrenergic receptor (β-AR) agonists activate the uncoupling protein (UCP) which causes the expenditure of metabolic calories as heat, is under pre-clinical stages [39]. Hormone-sensitive lipase (HSL) inhibitor improves lipid profiles and elevated insulin sensitivity (reduced plasma glucose levels) [40], is under pre-clinical stages. GPR40/ (Free fatty acid receptor 1 (FFAR1) ligand) regulates the secretion of glucagon- like peptide in the intestine, as well as increases insulin sensitivity41 Chronic exposure impairs β-cell function (lipotoxicity) (fasiglifam TAK-875 phase- III stage-discontinued) [42].

## Conclusion:

There are different families of oral and injectable drugs are available in the market for the treatment of T2DM. Considering the properties of the treatment such as effectiveness and durability of lowering blood Glucose, risk of hypoglycemia, diabetes complications, the effect on body weight, Side effects and contraindications, we need to develop a novel, safety and effective agents that will improve the quality of life of T2DM patients.
